# Effects of different concentrations of intraluminal sodium chloride solution on intracavitary ECG used for arm infusion port implantation

**DOI:** 10.1038/s41598-022-15156-z

**Published:** 2022-08-15

**Authors:** Lei Dong, Chen-yang Guan, Ying Zhang, Ai-xia Wang, Ming-hua Liu, Chen Guo, Xiao-li Hao, Qi Zhang

**Affiliations:** 1 Five Ward of Oncology Department, The First Affiliated Hospital of Zhengzhou University, Number One, Constructive East Road, Erqi District, Zhengzhou, 450052 China; 2 Second Department of Internal Medicine, The First Affiliated Hospital of Zhengzhou University, Zhengzhou, 450052 China

**Keywords:** Diseases, Oncology

## Abstract

At present, there are few clinical studies on the application of high-concentration sodium chloride solutions in intracavitary ECG-guided catheter tip localization during the arm infusion port implantation. This study observed the effects of sodium chloride solutions with different concentrations on intracavitary ECG-guided arm infusion port implantation in the patients with cancer. The 657 patients receiving arm infusion port implantation in our hospital between January 2020 and August 2021 were randomly divided into 0.9% sodium chloride solution conduction group (group A), 5.45% sodium chloride solution conduction group (group B) and 10% sodium chloride solution conduction group (group C). The derived rate of stable intracavitary ECG, the occurrence rate of characteristic P wave, the time used for catheter tip localization and the optimal position rate of catheter tip were compared between the three groups. The derived rate of stable intracavitary ECG was significantly higher in the group B (97.78%) and group C (98.63%) than in the group A (93.90%) (all *P* < 0.05). The occurrence rate of characteristic P wave was also significantly higher in the group B (96.89%) and group C (97.72%) than in the group A (88.73%) (all *P* < 0.001). The time used for catheter tip localization was significantly shorter in the group B [(49.73 ± 8.15) s] and group C [(48.27 ± 8.61) s] than in the group A [(69.37 ± 19.99) s] (all *P* < 0.001). There was no significant difference in the optimal position rate of catheter tip among the three groups (*P* > 0.05). The 5.45% and 10% sodium chloride solutions are significantly superior comparing with 0.9% sodium chloride solution in the derived rate of stable intracavitary ECG, occurrence rate of characteristic P wave and time used for catheter tip localization, but there were no significant differences between 5.45 and 10% sodium chloride solutions. Moreover, the 5.45% sodium chloride solution is closer to physiological state comparing with 10% sodium chloride solution, so the 5.45% sodium chloride solution may be recommended for the intracavitary ECG-guided arm infusion port implantation.

## Introduction

Arm infusion port is an implanted central vein pathway by inserting a catheter from the basilic vein or brachial vein. The arm infusion port can satisfy complex intravenous therapies, such as chemotherapeutic drug infusion, parenteral nutrition and hematopoietic stem cell transplantation^1^. It also has some advantages, over external catheters such as peripherally inserted central catheters, including beautiful appearance, less maintenance needs and prolonged indwelling time, so it has been widely used in the patients with cancer^2^. The optimal indwelling position of the central catheter tip is located in low one-third of the superior vena cava or at the intersection of the superior vena cava and right atrium^3^. If the tip position is overlength, the heart sensor is easily stimulated, resulting in chest tightness, arrhythmia and other complications. If the tip position is too shallow, tip drift easily occurs^4^. The arm infusion port implantation requires making a sac and sewing up an incision, so the accurate localization for the catheter tip may avoid re-opening the incision for adjusting the too deep or too shallow catheter tip position.

The American Infusion Nursing Society (INS) recommends that intracavitary ECG (IC-ECG) may be used to guide the catheter tip localization^3^. In clinical practice, the derived rates of stable intracavitary ECG and characteristic P wave are low when 0.9% sodium chloride solution or wire is used for electric conduction^5^. High-concentration sodium chloride solutions may obtain more stable IC-ECG than the 0.9% sodium chloride solution^6^. At present, there are few clinical studies on the application of high-concentration sodium chloride solutions in intracavitary ECG-guided catheter tip localization during the arm infusion port implantation, and the relevant guidelines do not give specific suggestions about concentration of sodium chloride solution for arm infusion port implantation. Therefore, this study observed the effects of sodium chloride solutions with different concentrations on intracavitary ECG-guided arm infusion port implantation, providing a basis for accurate catheter tip localization during the arm infusion port implantation.

## Materials and methods

All study methods were approved by the Ethics Committee of the First Affiliates Hospital of Zhengzhou University (2020-KY-0099), and were performed in accordance with relevant guidelines and regulations. All the subjects enrolled into the study gave written informed consent to participate.

### Subjects

According to a random number table, the patients receiving arm infusion port implantation in our hospital from January 2020 to August 2021, were divided into three groups, includng 0.9% sodium chloride solution conduction group (group A), 5.45% sodium chloride solution conduction group (group B) and 10% sodium chloride solution conduction group (group C). Inclusion criteria were (1) no contraindications for arm infusion port implantation, such as hemorrhagic tendency, bacteriemia, and being allergic to silicone, rubber or titanium alloy used in the arm infusion port implantation^7,8^; (2) clear consciousness; (3) age 18 years or older; and (4) surface ECG showing a normal P wave and sinus rhythm. Exclusion criteria were (1) arrhythmia or abnormal P wave, such as implanting heart pacemaker and atrial fibrillation; (2) psychiatric illness with poor compliance; (3) hypernatremia or hypokalemia; (4) a history of indwelling catheter or infusion port implantation; and (5) permanent left superior vena cava. A total of 657 patients were enrolled in this study and their general data are shown in Table 1.

**Table 1:** General data of patients in the three groups.

Items	Group A (n = 213)	Group B (n = 225)	Group C (n = 219)	*t*/χ^2^	*P* values
Age (year, ${\overline{\text{X}}}$ ± s)	47.43 ± 16.71	51.27 ± 12.89	50.67 ± 14.97	0.95	0.39
**Sex [n (%)]**				0.07	0.97
Men	117 (54.93%)	121 (53.78%)	118 (53.88%)		
Women	96 (45.07%)	104 (46.22%)	101 (46.12%)		
Diseases				13.80	0.09
Lymphoma	136 (63.85%)	143 (63.56%)	132 (60.27%)		
Lung cancer	38 (17.84%)	24 (10.67%)	40 (18.27%)		
Esophageal cancer	15 (7.04%)	17 (7.55%)	8 (3.65%)		
Gastric cancer	9 (4.23%)	20 (8.89%)	16 (7.31%)		
Others	15 (7.04%)	21 (9.33%)	23 (10.50%)		
Body height (cm, ${\overline{\text{X}}}$ ± s)	166.08 ± 7.64	168.63 ± 7.37	166.76 ± 7.83	1.27	0.28
Body weight (Kg, ${\overline{\text{X}}}$ ± s)	64.20 ± 10.77	66.01 ± 11.78	63.88 ± 12.16	0.43	0.65
BMI (Kg/m^2^, ${\overline{\text{X}}}$ ± s)	23.16 ± 2.72	23.20 ± 3.72	22.85 ± 3.17	0.17	0.84
Arm circumference (cm, ${\overline{\text{X}}}$ ± s)	26.86 ± 2.96	26.65 ± 2.65	26.99 ± 2.81	0.21	0.81
**Port arm [n (%)]**				1.08	0.58
Left	23 (10.80%)	26 (11.56%)	19 (8.68%)		
Right	190 (89.20%)	199 (88.44%)	200 (91.32%)		
**Punctured vein [n (%)]**				1.08	0.58
Basilic vein	174 (81.69%)	192 (85.33%)	184 (84.02%)		
Brachial vein	39 (18.31%)	33 (14.67%)	35 (15.98%)		
Implanted length (cm, ${\overline{\text{X}}}$ ± s)	37.69 ± 3.20	37.89 ± 2.42	37.22 ± 2.66	0.89	0.42
**Number of venous punctures**				0.90	0.92
Once	200 (93.90%)	214 (95.11%)	204 (93.15%)		
Twice	9 (4.23%)	8 (3.56%)	10 (4.57%)		
Three times or more	4 (1.87%)	3 (1.33%)	5 (2.28%)		

*BMI* body mass index.

### Materials

Implantable access ports were purchased from B. BRAUN MEDICAL (CHASSENEUIL, France). The port puncture diaphragm was made of silicone, and the catheter was made of 4.5Fr polyurethane. The ECG instrument (ECG-1350P) was purchased from Japan Optoelectronics Industry, Ltd (Tokyo, Japan). Color ultrasonic Doppler instrument (BA-50) was purchased from Bard Inc (New Jersey, USA).

### Methods

*Preparation of 5.45% sodium chloride solution* We used 10 ml of 0.9% sodium chloride solution to mix with 10 ml of 10% sodium chloride solution.

*Intracavitary ECG-guided catheter tip localization* The catheter end was connected with a heparin cap, then an injection needle was inserted into the heparin cap followed by connecting the alligator clip at one end of a sterile wire with the injection needle, and connecting the alligator clip at another end of a sterile wire with the metal buckle on the electrode slice placed on the right upper limb. Sodium chloride solution was intravenously injected (1–2 ml of 0.9% sodium chloride solution in group A, 1–2 ml of 5.45% sodium chloride solution in group B, and 1–2 ml of 10% sodium chloride solution in group C) until stable ECG occurred. The optimal indwelling position of the central catheter tip is located in low one-third of the superior vena cava or at the intersection of the superior vena cava and right atrium. When the catheter tip is located in the optimal indwelling position, P wave reaches its peak^9^. Therefore, the position of the catheter tip was adjusted according to the P wave. After the catheter entered, with the increase in the catheter entering depth, the P wave rose. When the P wave amplitude reached 50–80% of QRS wave amplitude, we paid close attention to the change of P wave. Once the P wave become low or diphasic, the catheter was withdrawn to the highest peak of the P wave, the ECG and the catheter implantation length were recorded.

Finally, all patients took chest X-ray examination to verify the accuracy of intracavitary ECG-guided catheter tip localization.

All the arm infusion port implantation was performed by 2 PICC nurses and one doctor in the venous catheterization room of our hospital. The two PICC nurses, one operator and one assistant, were responsible for intravenous catheter implantation. The operator had 14 years of clinical experience and the assistant had 8 years of clinical experience in central venous access implantation. The doctor was responsible for the port seat implantation and has been in this job for two years. All the 3 operators underwent and passed the electrocardiogram knowledge training. The doctor and assistant interpreted ICECG. Two radiologists interpreted chest X-rays and they engaged in this job for more than 10 years.

### Evaluations

*Stable ECG* The ECG baseline was stable, the P wave and QRS complex were clear, and their amplitudes were readable.

*Characteristic P wave*^5^ The P wave varied with adjusting the catheter tip position and the P wave had the highest amplitude in the ECG. When the catheter tip is located at the intersection of the superior vena cava and right atrium, the P wave is its highest amplitude^10^.

*Time used for catheter tip localization* It referred to the time from the catheter inserted 20 cm into the vein to the catheter tip placed at the optimal indwelling position.

*Optimal position rate of the catheter tip* The optimal position was that the catheter tip reached T6-T7 level.

### Statistical analysis

Statistical analysis was performed using SPSS21.0 software. Measurement data were expressed as mean ± standard deviation (X ± s) and underwent analysis of variance. Numeration data were expressed as frequency (n) or percentage (%), and underwent χ^2^ test. Statistical significance was established at *P* < 0.05.

## Results

### The derived rate of stable intracavitary ECG

Of the 657 patients, the stable ECG was not obtained from 21. There were significant differences in the derived rate of stable intracavitary ECG among the three groups (χ^2^ = 8.38, *P* < 0.05). The derived rate of stable intracavitary ECG was significantly higher in the group B (χ^2^ = 4.31, *P* < 0.05) and group C (χ^2^ = 7.27, *P* < 0.05) than in the group A. There was no significant difference in the derived rate of stable intracavitary ECG between the group B and group C (χ^2^ = 0.46, *P* > 0.05) (Table 2).

**Table 2:** The derived rate of stable intracavitary ECG and occurrence rate of characteristic P wave in the three groups [n (%)].

Groups	Stable ECG	Characteristic P wave
Group A (n = 213)	200 (93.90%)	189 (88.73%)
Group B (n = 225)	220 (97.78%)*	218 (96.89%)*
Group C (n = 219)	216 (98.63%)*	214(97.72%)*
*χ*^2^ values	8.38	19.06
*P* values	0.02	< 0.001

*Indicates *P* < 0.05 as compared with group A.

### The occurrence rate of characteristic P wave

Of the 657 patients, the characteristic P wave was not obtained from 36. This suggested that there was a stable ECG but no the characteristic P wave in 15 patients. There were significant differences in the occurrence rate of characteristic P wave among the three groups (χ^2^ = 19.06, *P* < 0.001). The occurrence rate of characteristic P wave was significantly higher in the group B (χ^2^ = 11.60, *P* < 0.001) and group C (χ^2^ = 15.01, *P* < 0.001) than in the group A. There was no significant difference in the occurrence rate of characteristic P wave between the group B and group C (χ^2^ = 0.29, *P* > 0.05) (Table 2).

### Time used for catheter tip localization

The time used for catheter tip localization was (69.37 ± 19.99) seconds in group A, (49.73 ± 8.15) seconds in group B and (48.27 ± 8.61) seconds in group C, respectively. There were significant differences in the time used for catheter tip localization among the three groups (*F* = 35.04, *P* < 0.001). The time used for catheter tip localization was significantly shorter in the group B (*P* < 0.001) and group C (*P* < 0.001) than in the group A. There was no sgnificant difference in the time used for catheter tip localization between group B anf group C (*P* = 0.63).

### Optimal position rate of the catheter tip

In this study, sodium chloride solution was used as electric conduction to successfully derive both stable ECG and characteristic P wave in 621 patients. Among the 621 patients, the chest X-ray examination indicated that the catheter tip was not at the optimal position in 14 patients. The distribution of catheter tip localization is shown in Table 3. There was not significantly different in the optimal position rate of catheter tip among the three groups (χ^2^ = 2.23, *P* > 0.05) (Table 3).

**Table 3:** The distribution of catheter tip localization in the three groups [n (%)].

Groups	T4-T5	T6-T7	T8-T9
Group A (n = 189)	1 (0.53%)	184 (97.35%)	4 (2.12%)
Group B (n = 218)	2 (0.92%)	213 (97.70%)	3 (1.38%)
Group C(n = 214)	0 (0.00%)	210 (98.13%)	4 (1.87%)
*P* value	0.69		

In the 36 patients without the characteristic P wave, wire-conductive method was used in them, as well as both stable ECG and characteristic P wave were derived from 27 patients, other 9 patients still had neither the stable ECG nor characteristic P wave. Finally, the length of catheter implantation was determined by electrocardio guidance combined with Rountree measurement^11^ in the 9 patients.

### Side effects

No uncomfortable symptoms, such as palpitation, chest tightness and general malaise occurred in all patients.

## Discussion

The European guidelines recommend real-time tip positioning in the central venous catheter^12^. This may avoid the risk of catheter malposition, reduce patients' and medical staff' exposures to X-ray, and save time and cost. IC-ECG positioning technology is simple, economical and radiation-free, and may be used in intraoperative positioning^13^.

The premise of successful IC-ECG positioning is to obtain a stable ECG, but an unstable and/or unclear ECG may appear due to interferences from some factors. In this study, 5.45% and 10% sodium chloride solutions were used as conductive mediators. Our results indicated that both derived rate of stable intracavitary ECG and occurrence rate of characteristic P wave were significantly higher in the 5.45% and 10% sodium chloride solutions than in the 0.9% sodium chloride solution. This may be because high concentration sodium chloride solutions had better electronic conductivities^14^. The high concentrations of Na(+) and Cl(−) can form a more stable conductive column, which enhances ECG signal and resists the influence of environmental signal, more easily obtaining stable ECG and characteristic P wave^15^.

In this study, the characteristic P wave was not obtained from 36 patients. This may be that the connection between the electrodes and the injection needle affected signal conduction^16^, or the signals transmitted by sodium chloride solution were incoherent, so a more stable conduction was established after using a guidewire for electric conduction. In clinical practice, in order to avoid the damage from the guidewire to the intima, the guidewire has to be withdrawn 0.5–1.0 cm, this affects the judgment for the catheter tip position to some extent. Moreover, in the guidewire conduction method, the withdrawn wire needs to be inserted into the catheter again, increasing the infection risk. Therefore, the sodium chloride solution as electric conduction should be the first choice for ECG-guided catheter tip localization. In this study, 636 patients obtained the stable ECGs, but 621 patients obtaine the characteristic P waves, indicating that 15 patients had the stable ECG but did not have the characteristic P wave. Pittiruti et al. have reported that the characteristic P wave is not able to derive in about 7% of patients with stable ECGs^17^. This potential cause remains to be further expolred.

In this study, the time used for catheter tip localization was significantly longer in the 0.9% sodium chloride solution than in the 5.45% and 10% sodium chloride solutions. This may be that the poor conductivity of the 0.9% sodium chloride solution required repeatedly injecting sodium chloride, which made the ECG wavetype unstable and affected the nurses' judgments for the catheter tip position^18^. However, 5.45% and 10% sodium chloride solutions could form a stable conductive column and derive more continuous and stable IC-ECG, so nurses could quickly judge the catheter tip position according to characteristic P wave and shortened the time used for catheter tip localization.

In the 621 patients who obtained the characteristic P waves in this study, the optimal position rate of the catheter tip was not significantly deferent among the 0.9%, 5.45% and 10% sodium chloride solutions, suggesting that the optimal position rate of the catheter tip was not related to the concentration of sodium chloride solution. The chest X-ray examination indicated that the catheter tip was not at the optimal position in 14 patients. This may be that the body position was not consistent between the chest X-ray examination and IC-ECG localization^19–21^.

The 5.45% and 10% sodium chloride solutions were superior to the 0.9% sodium chloride solution in the derived rate of stable IC-ECG, occurrence rate of characteristic P wave and time used for catheter tip localization, so high-concentration sodium chloride solutions (5.45% or 10%) were the first choice for the ECG-guided arm infusion port implantation. However, the 5.45% and 10% sodium chloride solutions were also not significantly different in the optimal position rate of catheter tip, and the 5.45% sodium chloride solution is closer to physiological concentration, so the 5.45% sodium chloride solution is the best choice for the ECG-guided arm infusion port implantation.

In addition, in this study, all patients in the three groups had no uncomfortable symptoms, such as palpitation and chest tightness. This may be that firstly the high-concentration sodium chloride solutions (5.45% or 10%) could obtain more stable signals and avoid repeatedly injecting sodium chloride, secondly the injected sodium chloride was rapidly diluted by the central venous blood flow and was not enough to stimulate the heart.

In this study, high-concentration sodium chloride solutions as conductive media achieved better results of catheter tip localization than 0.9% sodium chloride solution. We will further verify these results by larger sample size and multicentric studies.

### Limitations

Firstly, this study only investigated whether different concentrations of sodium chloride solutions could induce the lumen ECG and whether it could be used for catheter tip positioning. We did not record the amount of the sodium chloride solutions injected into intravenous injection. Secondly, the study period during which the patients were included into this study was short in this study. Thirdly, the patients of this study were only from a single center. We will carry out a large-sample, multicenter and randomized controlled study to further verify the accuracy and safety of different concentrations of sodium chloride solutions in arm infusion port implantation.

## Conclusions

The 5.45% and 10% sodium chloride solutions are significantly superior comparing with 0.9% sodium chloride solution in the derived rate of stable intracavitary ECG, occurrence rate of characteristic P wave and time used for catheter tip localization, but there were no significant differences between 5.45 and 10% sodium chloride solutions. Moreover, the 5.45% sodium chloride solution is closer to physiological state comparing with 10% sodium chloride solution, so the 5.45% sodium chloride solution is recommended for the intracavitary ECG-guided arm infusion port implantation.

## Data Availability

The datasets used and/or analysed during the current study available from the corresponding author on reasonable request.
